# Efficacy of international web‐based educational intervention in the detection of high‐risk flat and depressed colorectal lesions higher (CATCH project) with a video: Randomized trial

**DOI:** 10.1111/den.14244

**Published:** 2022-03-14

**Authors:** Mineo Iwatate, Daizen Hirata, Carlos Paolo D. Francisco, Jonard Tan Co, Jeong‐Sik Byeon, Neeraj Joshi, Rupa Banerjee, Duc Trong Quach, Than Than Aye, Han‐Mo Chiu, Louis H. S. Lau, Siew C. Ng, Tiing Leong Ang, Supakij Khomvilai, Xiao‐Bo Li, Shiaw‐Hooi Ho, Wataru Sano, Santa Hattori, Mikio Fujita, Yoshitaka Murakami, Masaaki Shimatani, Yuzo Kodama, Yasushi Sano, The CATCH project team

**Affiliations:** ^1^ Gastrointestinal Center and Institute of Minimally‐invasive Endoscopic Care (iMEC) Sano Hospital Hyogo Japan; ^2^ Division of Gastroenterology Department of Internal Medicine Kobe University Graduate School of Medicine Hyogo Japan; ^3^ Department of Medical Statistics Toho University Tokyo Japan; ^4^ The Third Department of Internal Medicine Division of Gastroenterology and Hepatology Kansai Medical University Medical Center Osaka Japan; ^5^ Department of Gastroenterology and Hepatology Kindai University Osaka Japan; ^6^ Kansai Medical University Osaka Japan; ^7^ Institute of Digestive and Liver Diseases St. Luke’s Medical Center Taguig City Philippines; ^8^ Department of Gastroenterology Asan Medical Center University of Ulsan College of Medicine Seoul Korea; ^9^ Gastro Enterology Unit Nepal Cancer Hospital and Research Centre Lalitpur Nepal; ^10^ Medical Gastroenterology Asian Institute of Gastroenterology New Delhi India; ^11^ University of Medicine and Pharmacy at Ho Chi Minh City Ho Chi Minh Vietnam; ^12^ 249348 University of Medicine 2 Yangon Myanmar; ^13^ Department of Internal Medicine National Taiwan University Hospital Taipei Taiwan; ^14^ Department of Medicine and Therapeutics Faculty of Medicine Institute of Digestive Disease The Chinese University of Hong Kong Hong Kong China; ^15^ Division of Gastroenterology and Hepatology Key Laboratory of Gastroenterology and Hepatology Ministry of Health, Renji Hospital School of Medicine Shanghai Institute of Digestive Disease Shanghai Jiao Tong University Shanghai China; ^16^ Department of Gastroenterology and Hepatology Changi General Hospital SingHealth Singapore; ^17^ Surgical Endoscopy Colorectal Division Department of Surgery Faculty of Medicine Chulalongkorn University Bangkok Thailand; ^18^ Department of Medicine Faculty of Medicine University of Malaya Kuala Lumpur Malaysia

**Keywords:** colonoscopy, detection, education, flat and depressed lesions, randomized trial

## Abstract

**Objectives:**

Three subcategories of high‐risk flat and depressed lesions (FDLs), laterally spreading tumors non‐granular type (LST‐NG), depressed lesions, and large sessile serrated lesions (SSLs), are highly attributable to post‐colonoscopy colorectal cancer (CRC). Efficient and organized educational programs on detecting high‐risk FDLs are lacking. We aimed to explore whether a web‐based educational intervention with training on FIND clues (fold deformation, intensive stool/mucus attachment, no vessel visibility, and demarcated reddish area) may improve the ability to detect high‐risk FDLs.

**Methods:**

This was an international web‐based randomized control trial that enrolled non‐expert endoscopists in 13 Asian countries. The participants were randomized into either education or non‐education group. All participants took the pre‐test and post‐test to read 60 endoscopic images (40 high‐risk FDLs, five polypoid, 15 no lesions) and answered whether there was a lesion. Only the education group received a self‐education program (video and training questions and answers) between the tests. The primary outcome was a detection rate of high‐risk FDLs.

**Results:**

In total, 284 participants were randomized. After excluding non‐responders, the final data analyses were based on 139 participants in the education group and 130 in the non‐education group. The detection rate of high‐risk FDLs in the education group significantly improved by 14.7% (66.6–81.3%) compared with −0.8% (70.8–70.0%) in the non‐education group. Similarly, the detection rate of LST‐NG, depressed lesions, and large SSLs significantly increased only in the education group by 12.7%, 12.0%, and 21.6%, respectively.

**Conclusion:**

Short self‐education focusing on detecting high‐risk FDLs was effective for Asian non‐expert endoscopists. (UMIN000042348).

## Introduction

Colonoscopy can reduce the incidence and mortality of colorectal cancer (CRC) by detecting and removing precursor lesions.^1^ Flat and depressed lesions (FDLs) are more easily overlooked during colonoscopy and result in post‐colonoscopy CRC (PCCRC) due to their subtle appearance and a higher risk of advanced histology with subsequent malignant transformation.^2^, ^3^ The detection of FDLs is worthy of attention given that their proportion among all neoplasia was almost similar between the West (14.8–37.6%) and the East (6.2–42.0%) populations.^4^, ^5^, ^6^, ^7^, ^8^, ^9^, ^10^

Three subcategories of FDLs having a more aggressive nature can be at high risk for PCCRC. First, depressed lesions are considered the most rapid growth in all macroscopic types.^11^, ^12^, ^13^ Their submucosal invasion rates even in diminutive and small polyps were high (8.4–43.6%) compared with the other types (0–1.3%).^3^ Second, laterally spreading tumors non‐granular type (LST‐NG) was most likely missed during colonoscopy, accounting for over 80% of incident advanced FDLs detected even after a two‐round baseline colonoscopy by experienced endoscopists.^14^ Reportedly, both LST‐NG and depressed lesions were associated with 71% of early‐stage PCCRC and 67% of metachronous early‐stage CRC at the first surveillance after surgical resection for initial CRC.^15^, ^16^ Third, sessile serrated lesions (SSLs) can also be missed because of their flat morphology and rapid progression into invasive cancer once dysplasia develops.^17^, ^18^, ^19^ Reportedly, two‐thirds of SSL‐associated cancers are estimated to be interval malignancies of the proximal colon.^20^ As the size increases, SSLs contain a higher proportion of dysplasia, reaching 13.6% in large (≥10 mm) SSLs.^21^ Collectively, it is reasonable to speculate that three subcategories of FDLs (LST‐NG, any depressed lesions, and large SSLs) are highly attributable to PCCRC.

In this context, we concluded that training on detecting three subcategories of high‐risk FDLs is in high demand, and the presence of the learning curve would enhance its importance.^22^ Regarding its clinical significance in preventing PCCRC, the current real‐time computer‐aided detection (CADe) system failed to improve the detection of LSTs and SSLs during colonoscopy.^23^ Kaminski *et al*. reported that leadership training (assessment, hands‐on training, post‐training feedback) significantly improved the adenoma detection rate and FDL detection rate.^24^ However, efficient and organized educational programs specific for the detection of high‐risk FDLs are lacking.

**Figure 1:**
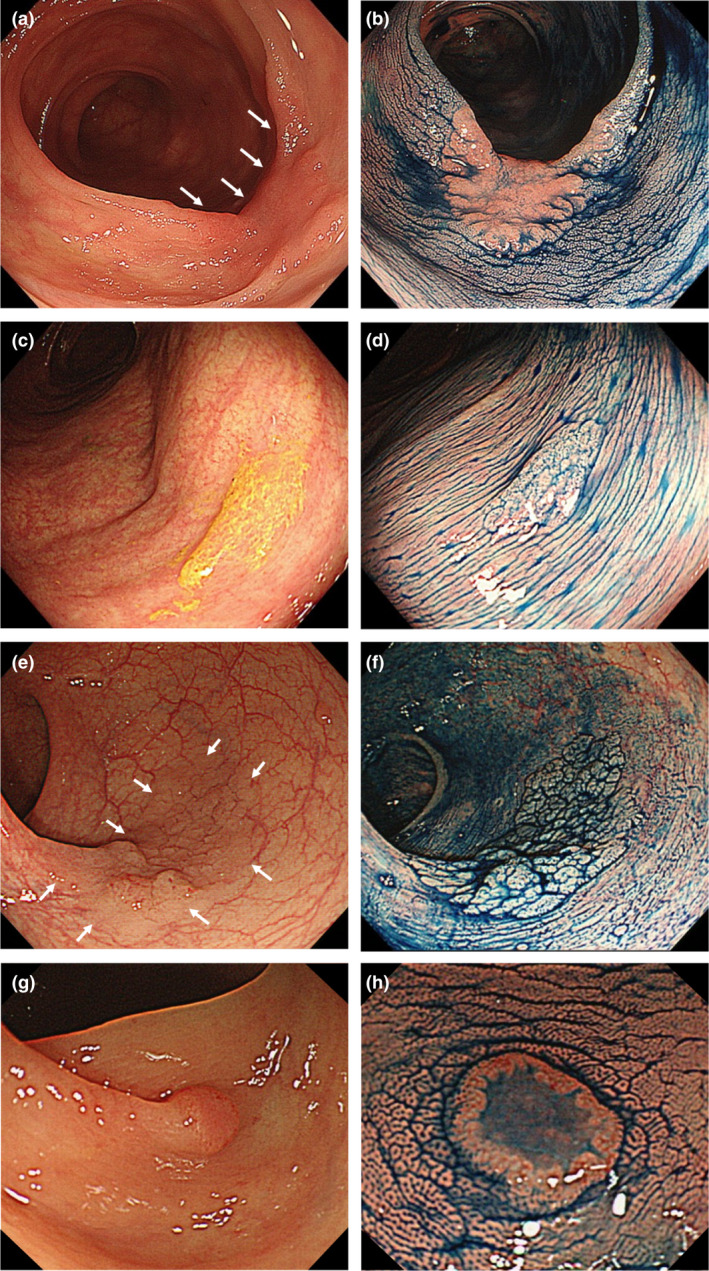
The FIND clues. (a) Fold deformation (white arrows). Laterally spreading tumors non‐granular type (LST‐NG) on the fold forms this change. (b) Chromoendoscopy clearly demonstrates the LST‐NG 35 mm in size. (c) Intensive stool/mucus attachment. The mass of stools is accumulated. (d) After removing the stool and mucus, the sessile serrated lesion, 10 mm in size, is visualized by chromoendoscopy. (e) No vessel visibility (white arrows). A flat lesion masks the visibility of the vessels in the background mucosa. (f) Chromoendoscopy reveals LST‐NG 30 mm in size. (g) Demarcated reddish area. A small marginally reddish area (o‐ring sign) is recognized. (h) Depressed lesion 5 mm in size can be seen by chromoendoscopy.

We propose the four key clues, “FIND”: Fold deformation, Intensive stool/mucus attachment, No vessel visibility, and Demarcated reddish area (Fig. 1) under white‐light endoscopy to help detect the high‐risk FDLs. We considered that catching the key clues for high‐risk FDLs was much easier and more practical than recognizing their whole shape. The background of the FIND clues is as follows. (i) Fold deformation: LST‐NG is often located on the fold and forms fold deformation. (ii) Intensive stool mucus attachment: SSLs produce mucus, which traps stools on their surface.^25^ (iii) No vessel visibility: the FDLs mask the visibility of vessels in the background mucosa by themselves. (iv) Demarcated reddish area: the color of LST‐NG and depressed lesions are usually reddish compared with pale color in SSLs. The reddish color is classified into the whole reddish type, and the marginal reddish type called the o‐ring sign.^26^

This randomized trial aimed to examine whether self‐education focusing on FIND clues can improve the detection rate of high‐risk FDLs by non‐expert endoscopists in 13 Asian countries.

## Methods

### Study design

This was an international web‐based parallel randomized control study that enrolled non‐expert endoscopists in 13 Asian countries.

The study was conducted in four phases: (i) recruitment and randomization; (ii) pre‐test; (iii) education; and (iv) post‐test. The participants allocated either an education or a non‐education group were asked to take the pre‐test and post‐test on the web. Only the education group received a short self‐education program on recognizing the FIND clues between two tests. We compared the change in the detection rate of high‐risk FDLs between two groups.

The study was approved by the Institutional Review Board at Sano Hospital (202010‐2), and registered with the University Hospital Medical Information Network Clinical Trials Registry (UMIN000042348).

### Participants

We recruited endoscopists from 13 Asian countries between November 2020 and February 2021, including China, Hong Kong, India, Japan, Malaysia, Myanmar, Nepal, Philippines, Korea, Singapore, Taiwan, Thailand, and Vietnam. Each country has one local coordinator who supports the recruitment of the participants and encourages non‐responders to take the tests or education program. Inclusion criteria were: (i) clinical experience of endoscopy <10 years; (ii) take the test and watch the sample materials in English on the web; and (iii) provide written informed consent for participation in the study. Exclusion criteria were: (i) non‐completion of the pre‐test or the post‐test; and (ii) non‐completion of education program in the education group.

All participants were randomly assigned (1:1 ratio) to either the education or non‐education group using computer‐generated randomization with a minimization method (factors: country and experience of colonoscopy). None of the participants were informed about the study outcome measures.

### Selection of the test images

**Figure 2:**
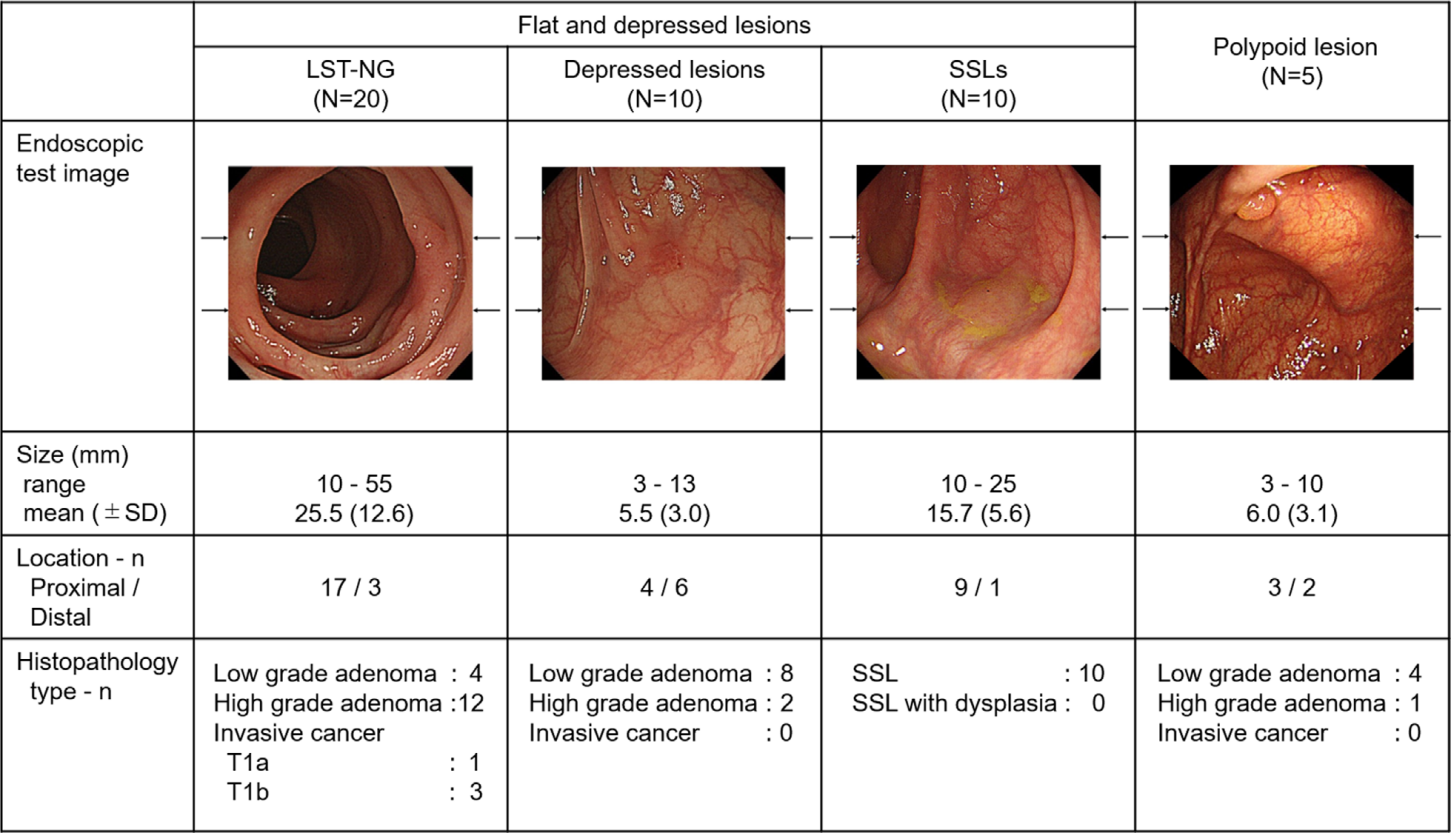
Baseline characteristics of colorectal lesions in the test images. LST‐NG, laterally spreading tumor non‐granular type; SD, standard deviation; SSL, sessile serrated lesion.

We randomly selected 60 still endoscopic high‐definition images taken by white‐light imaging from the endoscopic image library stored at Sano Hospital between 2006 and 2020. The test images were composed of 20 LST‐NG (size range 10–55 mm), 10 depressed (IIc) lesions (3–13 mm), 10 large SSLs (10–25 mm), five polypoid lesions (3–10 mm), and 15 had no lesions. Among FDLs, we included LST‐NG more than the other types, considering the actual prevalence of each FDL.^3^, ^27^, ^28^ With the black arrows on the outside of the image, each image was virtually divided into three areas (upper, middle, and lower) for readers to help understand the main area of the detected lesion in the image (Fig. 2). Six expert endoscopists discussed and confirmed the presence of a target lesion with its main occupied area (upper, middle, and lower) or no lesions in each test image, which was used as the correct answer for the test.

### Educational program

The web‐based education program involving an education video and self‐learning training took place only for the education group in May 2021 between the tests. First, all participants in the education group watched the 15‐min education video describing the clinical importance of the high‐risk FDLs and how to detect them using the FIND clues (Video S1). Then, they were required to take self‐learning training (20 questions and answers) about recognizing the FIND clues and their target lesions with a detailed clinical explanation (Figs S1,S2). This self‐learning training provided endoscopic images of high‐risk FDLs that were not used in the pre‐test or post‐test.

### Pre‐test and post‐test

All participants were asked to take both the pre‐test in March and the post‐test in June 2021 (the time duration between both tests was 2 months) on the web.

In the pre‐test phase, the participants read 60 still endoscopic images. They chose one answer from four options (there is no polyp in the image/there is a polyp in the upper area/the middle area/the lower area). To reduce accidentally choosing correct answers, the participants were required to select the main area of the detected lesion in the test. The reading time for each image was set to 5 s, considering that the time taken to detect polyps during colonoscopy is limited in clinical practice.

In the post‐test phase, all participants read the same 60 still endoscopic images used in the pre‐test and chose one answer in the same way as the pre‐test. Only the education group was also required to determine the usefulness of the FIND clues (multiple choice) for each image when they detected a polyp in the post‐test.

### Variables

For each image in the test, one score point was given when the participants selected the correct answer to calculate the detection rates of colorectal lesions.

Before the pre‐test, we collected the background data of the participants, including sex, age, country, clinical experience of colonoscopy (0–9 years), and the number of colonoscopies performed in their lives. We investigated the number of total endoscopists in each Asian country from local coordinators to calculate the proportion of endoscopists per population.

### Primary and secondary outcome measures

The primary outcome was the difference in the detection rates between the pre‐test and post‐test for FDLs (LST‐NG, depressed lesions, and large SSLs) in both groups.

The secondary outcomes were: (i) factors associated with increasing the detection rate (≥10%) for the FDLs; and (ii) the frequency of the FIND clues detected in the FDLs in the education group.

### Sample size calculation

A previous study by Yao *et al*. showed that the intervention (e‐learning) group could increase around 10% (score from 51.4 to 62.2 out of 100) of the detection rate of early gastric cancer than that of the non‐intervention group.^29^ Considering the control group would also increase scores if they memorized the test images and learned detection tips by themselves, we assumed the average changes in scores in both the intervention and control groups of 10 (=80–70) and 5 (=75–70), respectively. Two‐sided *t*‐tests with equal variance (power = 80%, alpha = 5%) were used in this study. We planned to compare the results from countries with high dissemination rates in colonoscopy practice and those with low dissemination rates. In each group, we finally chose 128 participants to include in this study (total number of doctors, 256). Based on this sample size, we decided to recruit 20 doctors from each country.

### Statistical analysis

We calculated the difference in scores before and after the intervention for each individual. The mean differences between the intervention and control groups were compared using a *t*‐test. We also conducted a multiple regression analysis to explore a person's characteristics of increasing scores. The variables examined were sex, age, years of experience, country, pre‐test scores, and education. The outcome measures used in the analysis were adjusted mean differences in multiple regression and adjusted odds ratios in logistic regression. The logistic regression was performed by dichotomizing the scores into high (score change of three or more) and low (score change of less than three) and included them into the model as an outcome. All statistical analyses were performed using the Statistical Analysis System (release 9.40; SAS, Cary, NC, USA). All statistical tests were two‐sided, and the significance level was set at 0.05.

## RESULTS

### Study population

**Figure 3:**
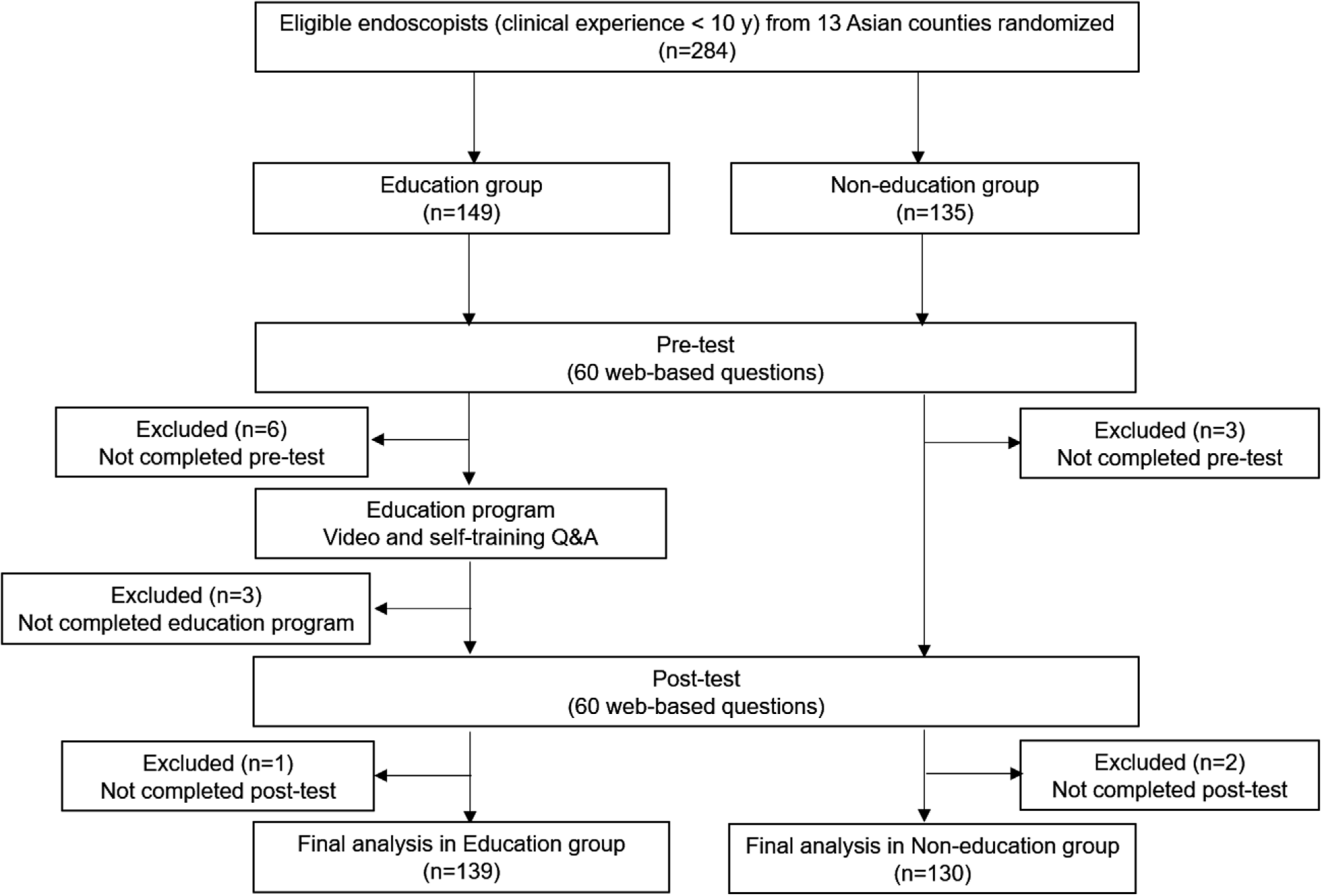
Study flowchart.

A total of 284 endoscopists who met the inclusion criteria were registered with a written informed consent form from 13 Asian countries. Of the 284 participants, 149 were allocated to the education group and 135 to the non‐education group. After excluding participants who did not complete at least one test or educational program, the final data analyses were conducted based on the 139 participants in the education group (follow‐up rate 93.3%) and 130 in the non‐education group (96.3%) (Fig. 3). The baseline characteristics of the participants in the education and non‐education groups were similar (Table 1). In the education group, the educational program took 40 min on average, including 15 min of watching the educational video and 25 min of self‐education training.

**Table 1:** Baseline characteristics of the participants

	Education group (*N* = 139)	Non‐education group (*N* = 130)	*P*‐value
Baseline characteristics			
Age, years; mean (±SD)	35.7 (4.6)	35.6 (4.7)	0.90
Male sex, *n* (%)	91 (65.5)	83 (63.8)	0.78
Colonoscopy experience, years; mean (±SD)	4.3 (2.5)	4.3 (2.3)	0.94
Number of colonoscopies performed, mean (±SD)	1521 (2733)	1675 (2596)	0.43
Country, *n*			
China	8	9	
Hong Kong	11	9	
India	11	11	
Japan	10	10	
Malaysia	9	6	
Myanmar	13	8	
Nepal	12	10	
Philippines	11	17	
Korea	13	10	
Singapore	13	7	
Taiwan	8	12	
Thailand	9	11	
Vietnam	11	10	

### Study outcomes

#### Change in detection rates between pre‐test and post‐test in both groups

As shown in Table 2, the detection rates for the FDLs in the education group significantly improved by 14.7% (from 66.6% to 81.3%) compared with −0.8% (from 70.8% to 70.0%) in the non‐education group. Similarly, the detection rates for the LST‐NG, depressed lesions, and large SSLs significantly improved only in the education group by 12.7%, 12.0%, and 21.6%, respectively. In contrast, a barely significant decrease in the detection rate for the polypoid lesion by 1.9% (from 98.4% to 96.5%) was found only in the education group. The detection rate for the no lesion significantly deteriorated by 11.1% (from 84.4% to 73.3%) in the education group but increased by 3.7% (from 79.8% to 83.5%) in the non‐education group.

**Table 2:** Change in detection rates between pre‐test and post‐test in both groups and education effects

	Education group (*N* = 139)			Non‐education group (*N* = 130)			Education effects	
	Pre‐test (%)	Post‐test (%)	*P*‐value	Pre‐test (%)	Post‐test (%)	*P*‐value	Difference in changes (%)^†^	*P*‐value
Flat and depressed lesions (40 cases)	66.6	81.3	<0.01	70.8	70.0	0.37	15.6	<0.01
LST‐NG (20 cases)	71.7	84.4	<0.01	74.9	73.5	0.19	14.1	<0.01
Depressed lesions (10 cases)	59.8	71.8	<0.01	63.5	64.9	0.37	10.6	<0.01
Large SSLs (10 cases)	63.2	84.8	<0.01	70.0	68.1	0.20	23.5	<0.01
Polypoid lesions (5 cases)	98.4	96.5	0.02	98.0	97.2	0.32	−1.1	0.32
No lesions (15 cases)	84.4	73.3	<0.01	79.8	83.5	0.02	−14.8	<0.01
All (60 cases)	73.7	80.6	<0.01	75.3	75.6	0.68	6.6	<0.01

^†^ Change in the detection rates in the education group minus that in the non‐education group.

LST‐NG, laterally spreading tumor, non‐granular type; SSL, sessile serrated lesion.

#### Factors associated with an increased detection rate (≥10%) for FDLs

Table 3 shows multiple logistic regression analyses to identify any significant factors for an increased detection rate (≥10%) for FDLs. The factors analyzed were sex, age, years of experience in colonoscopy, country, pre‐test score for FDLs, and education. The 13 Asian countries were divided into two groups based on the number of endoscopists per million population.

**Table 3:** Factors associated with an increased detection rates (≥10%) for the flat and depressed lesions

	Multivariate analysis		
	Estimate	95% confidence interval	*P*‐value
Sex male (Ref: female)	0.15	−0.97 to 1.27	0.79
Age	0.05	−0.08 to 0.19	0.43
Experience years of coloscopy	0.11	−0.15 to 0.37	0.42
Countries‐high endoscopist proportion^†^ (Ref: Countries‐low endoscopist proportion)	0.79	−0.36 to 1.94	0.18
Pre‐test score (unit: one score decrease)	0.41	0.33 to 0.48	<0.01
Education group (Ref: non‐education group)	5.51	4.44 to 6.58	<0.01

^†^ Countries‐high endoscopist proportion (19–167 endoscopists per million population); China, Hong Kong, Japan, Malaysia, Korea, Singapore, Taiwan. Countries‐low endoscopist proportion (1–10); India, Myanmar, Nepal, Philippines, Thailand, Vietnam.

The multivariate analysis demonstrated that the education group and the pre‐test score were significantly associated with an increased score for FDLs.

#### Frequency of FIND clues in FDLs

In the education group, the frequency of FIND clues in FDLs is summarized in Table 4. The fold deformation (69.4%) was the most frequently found in the LST‐NG, followed by no vessel visibility (53.2%). No vessel visibility (69.7%) was the most significantly associated with the SSL, followed by intensive stool/mucus attachment (37.6%). In the depressed lesion, the demarcated reddish area (49.7%) and the no vessel visibility (45.0%) were the most frequently found.

**Table 4:** Frequency of the FIND clues in the high‐risk flat and depressed lesions in the education group

	Frequency in LST‐NG (%) (*n* = 2780)	Frequency in depressed lesions (%) (*n* = 1390)	Frequency in large SSLs (%) (*n* = 1390)
Fold deformation	69.4	32.8	21.3
Intensive stool/mucus attachment	3.8	6.0	37.6
No vessel visibility	53.2	45.0	69.7
Demarcated reddish area	42.3	49.7	21.0

LST‐NG, laterally spreading tumor, non‐granular type; SSL, sessile serrated lesion.

The false‐positive rates of the FIND clues for the 15 no lesions were 8.6% for the fold deformation, 1.6% for the intensive stool/mucus attachment, 16.1% for the no vessel visibility, and 7.9% for the demarcated reddish area.

## Discussion

This is the first international randomized control trial to show that educational intervention by training on FIND clues effectively improved the detection of high‐risk FDLs. Our study had two strengths. First, we focused mainly on high‐risk flat and depressed neoplasms as the target lesions. The clinical impact on the detection of these target lesions is much greater than that of small flat lesions with low malignant potential.^3^ Second, we first developed a 40‐min self‐education program focusing on FIND clues. FIND clues based on simple white‐light findings are available without advanced technology and applied after short‐term education on the web.

**Figure 4:**
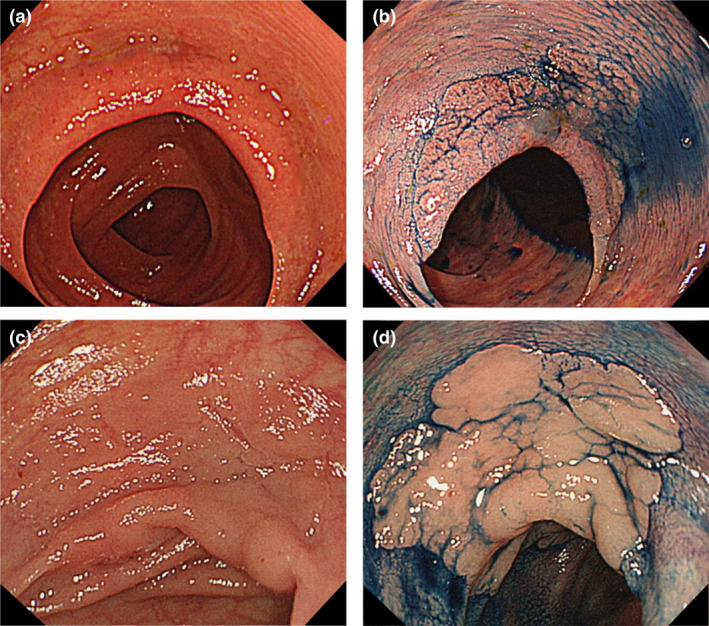
Extremely large laterally spreading tumors non‐granular type (LST‐NGs) missed in the test. (a) A large LST‐NG (50 mm) on the fold was missed by 39% (105/269) of the participants. (b) Chromoendoscopy reveals the whole shape of LST‐NG on the fold. Histopathology was high‐grade adenoma. (c) A large LST‐NG (55 mm) on the fold was missed by 17% (45/269) of the participants. (d) Chromoendoscopy makes the margin of the LST‐NG clear. Histopathology was deep submucosal invasive cancer (T1b).

Our primary study outcome was the change in the detection rates for high‐risk FDLs between the two groups. In the education group, the detection rates significantly improved for LST‐NG, depressed lesions, SSLs, and all high‐risk FDLs. The results of the multivariate analysis in Table 3 make our development more robust. It should be noted that even extremely large FDLs were missed. We found miss rates of the two extremely large (≥50 mm) LST‐NG were high (17% and 39%) in the pre‐test (Fig. 4). The detection rate of the polypoid lesion significantly decreased after education; however, that in the post‐test remained high at 96.5%. The significantly increased detection rate of no lesions in the education group, which is equivalent to “false positive” can cause prolonged withdrawal time in clinical practice. Because inflammations, insufficient pooled air in the colon, or poor preparation can also create the FIND clues, some participants would overdiagnose the FIND clues after the intervention. However, advanced modalities such as image‐enhanced endoscopy (IEE) can prevent unnecessary treatment for false lesions. In the non‐education group, the detection rate of no lesions was significantly elevated, probably because the lack of concentration in the post‐test without any intervention made the participants choose more no lesions. The fact that the average time to complete the same test reduced from 23 min in the pre‐test to 18 min in the post‐test for the non‐education group might support our speculation.

Previous studies showed that IEE significantly improved the detection of flat lesions; however, most were small and had low malignant potential.^30^, ^31^, ^32^, ^33^, ^34^, ^35^ In the era of rapidly evolving artificial intelligence technologies, the CADe system for detecting LSTs and SSLs achieved high sensitivity in studies using video materials; however, its advantage in real‐world colonoscopy remains elusive.^23^, ^36^ Even if the CADe system could detect high‐risk FDLs in real‐time colonoscopy, human‐eye detection and recognition are still essential because an actual lesion detected by the CADe system could be missed unless the endoscopist has sufficient experience to recognize it as a true lesion.^37^ The better interaction between CADe and human‐eye detection with the FIND clues is indispensable and will achieve the best performance in the future.

Our study has some limitations. First, we employed an *in vitr*o study design using standardized images to avoid inconsistencies in the diagnostic criteria of macroscopic type, especially LST‐NG, in each country. Further studies on identifying FIND clues in real clinical settings are necessary. Second, we focused mainly on large flat and small depressed lesions. Theoretically, the FIND clues should be applicable to small flat lesions; however, their effectiveness remains unclear in our trial. Third, the study participants were limited to Asian endoscopists. Considering that the prevalence of FDLs is similar between the West and the East, our education program is also applicable to Western endoscopists. Fourth, the lower pre‐test score in the education group might affect the high education effect on the detection rate of high‐risk FDLs.

In conclusion, short self‐education focusing on detecting high‐risk FDLs was effective for Asian non‐expert endoscopists. Learning FIND clues may contribute to decreasing PCCRC in the future.

## CONFLICT OF INTEREST

Author H.‐M.C. is an Associate Editor of *Digestive Endoscopy*. The other authors declare no conflict of interest for this article.

## FUNDING INFORMATION

This study was supported by the Asian Endoscopy Research Forum (AERF) research grant (2020/21).

## Supporting information


**Figure S1** (a–c) Self‐training question and answer (Case 1).
**Figure S2** 60 test images.

**Video S1** Education video for learning the FIND clues (15 min).
