# Supplementary figures and images for: Efficacy of international web‐based educational intervention in the detection of high‐risk flat and depressed colorectal lesions higher (CATCH project) with a video: Randomized trial
Source: Dig Endosc. 2022 Mar 14;34(6):1166–75. doi: 10.1111/den.14244 (PMC9540870; doi:10.1111/den.14244)

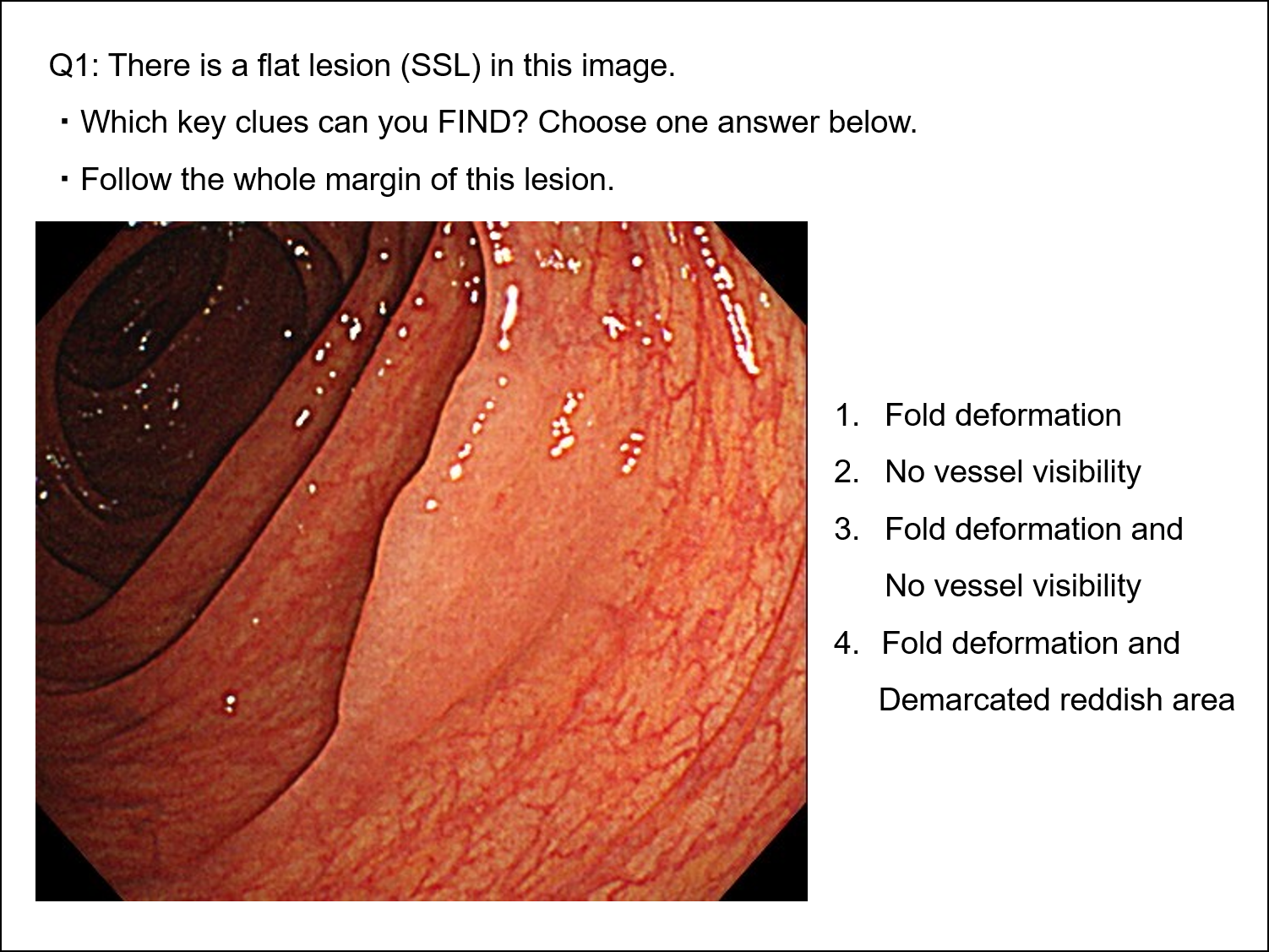

Supplement: Supplementary file 1 — Figure S1 (a–c) Self‐training question and answer (Case 1). Figure S2 60 test images. [file DEN-34-1166-s001.zip › den14244-sup-0001-FigS1A.tif]

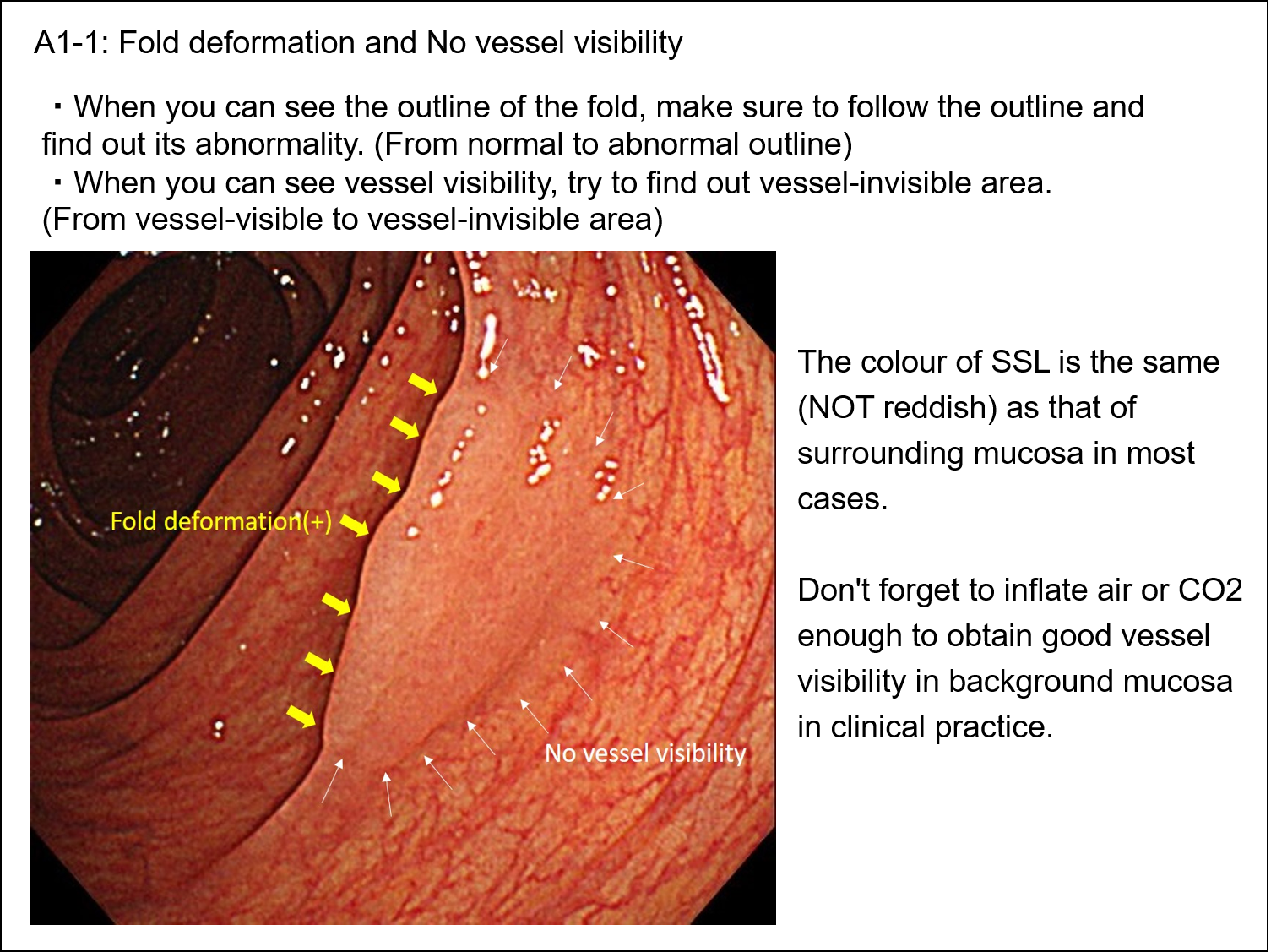

Supplement: Supplementary file 1 — Figure S1 (a–c) Self‐training question and answer (Case 1). Figure S2 60 test images. [file DEN-34-1166-s001.zip › den14244-sup-0002-FigS1B.tif]

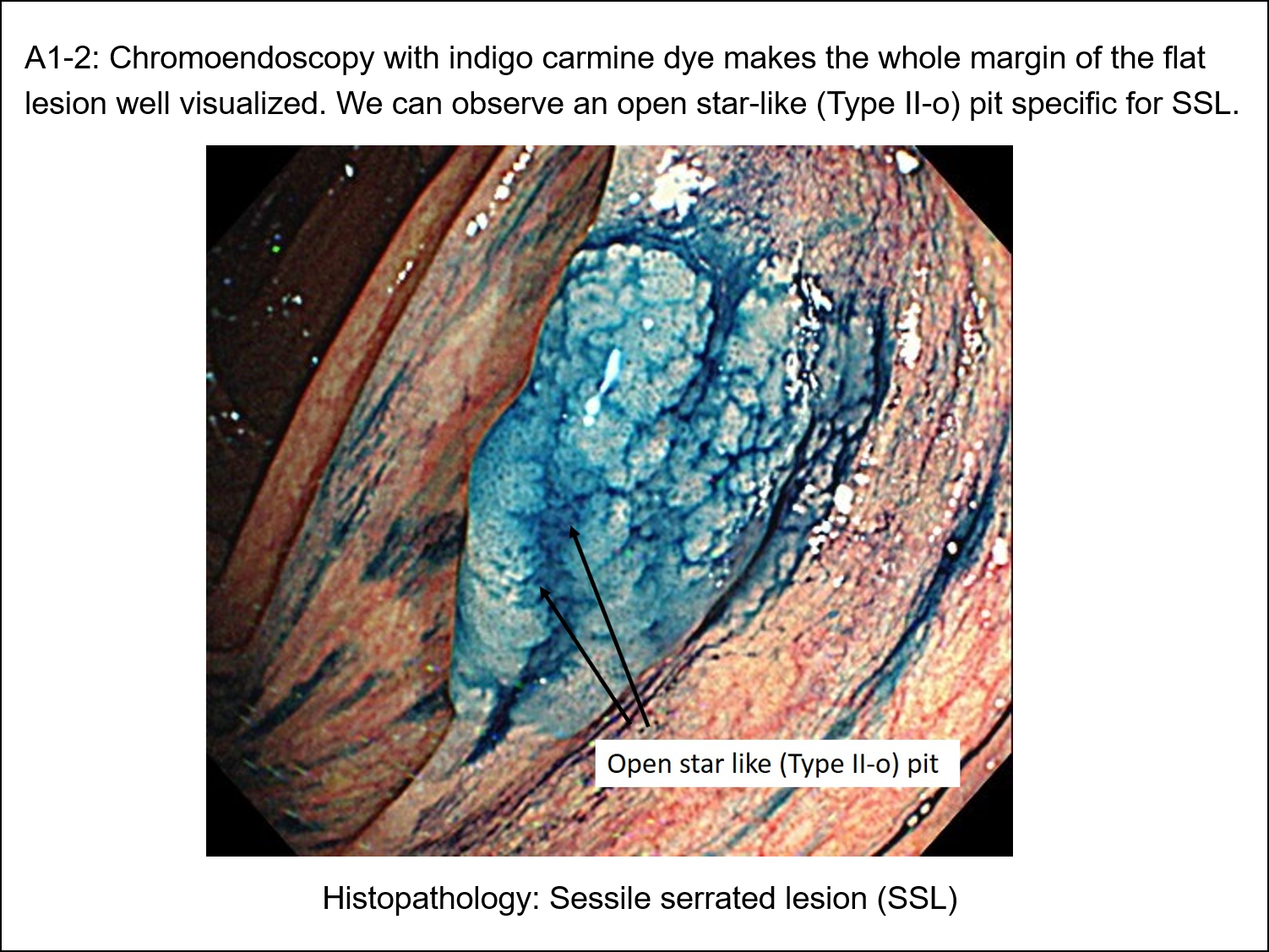

Supplement: Supplementary file 1 — Figure S1 (a–c) Self‐training question and answer (Case 1). Figure S2 60 test images. [file DEN-34-1166-s001.zip › den14244-sup-0003-FigS1C.tif]

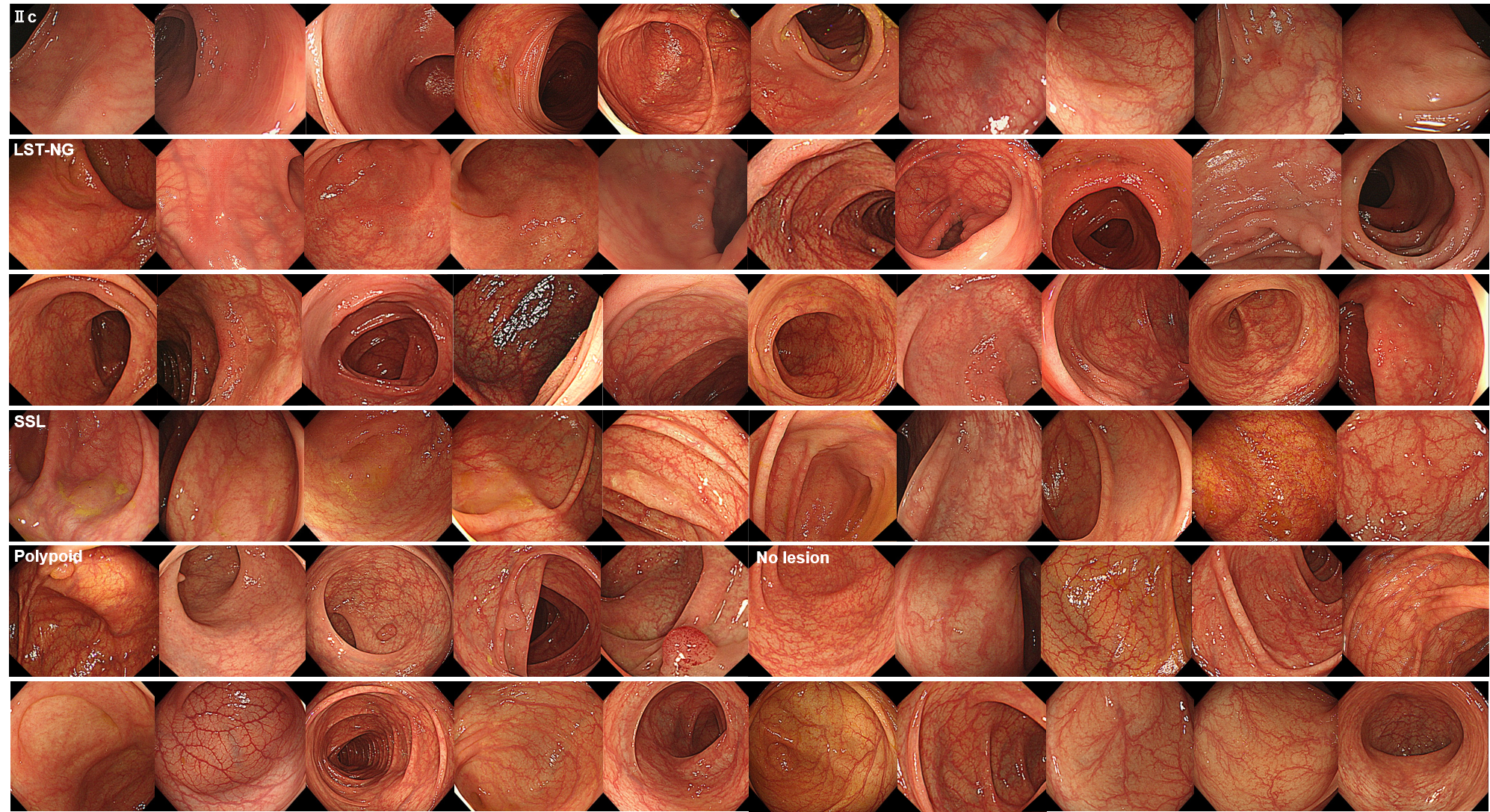

Supplement: Supplementary file 1 — Figure S1 (a–c) Self‐training question and answer (Case 1). Figure S2 60 test images. [file DEN-34-1166-s001.zip › den14244-sup-0004-FigS2.tif]
