# Supplementary material for: Efficacy of international web‐based educational intervention in the detection of high‐risk flat and depressed colorectal lesions higher (CATCH project) with a video: Randomized trial
Source: Dig Endosc. 2022 Mar 14;34(6):1166–75. doi: 10.1111/den.14244 (PMC9540870; doi:10.1111/den.14244)
Supplement: Supplementary file 2 [file DEN-34-1166-s002.docx]

**Video link**

**・Youtube**

<https://youtu.be/CciORX7AfZs>

**・Vimeo**

[**https://vimeo.com/617718577**](https://vimeo.com/617718577)
